# Scaffold-Dependent Mechanical and Architectural Cues Guide Osteochondral Defect Healing *in silico*

**DOI:** 10.3389/fbioe.2021.642217

**Published:** 2021-02-15

**Authors:** Martina Tortorici, Ansgar Petersen, Klara Ehrhart, Georg N. Duda, Sara Checa

**Affiliations:** ^1^Julius Wolff Institute, Charité Universitaetsmedizin Berlin, Berlin, Germany; ^2^Berlin-Branderburg School for Regenerative Therapies, Charité Universitaetsmedizin Berlin, Berlin, Germany; ^3^Berlin Institute of Health Center for Regenerative Therapies, Charité Universitaetsmedizin Berlin, Berlin, Germany; ^4^Continuum Mechanics and Material Theory, Faculty V of Mechanical Engineering and Transport Systems, Institute of Mechanics, Technische Universtitaet Berlin, Berlin, Germany

**Keywords:** osteochondral defect, tissue engineering, scaffold, computer model, mechanobiology

## Abstract

Osteochondral defects in joints require surgical intervention to relieve pain and restore function. However, no current treatment enables a complete reconstitution of the articular surface. It is known that both mechanical and biological factors play a key role on osteochondral defect healing, however the underlying principles and how they can be used in the design of treatment strategies remain largely unknown. To unravel the underlying principles of mechanobiology in osteochondral defect healing, i.e., how mechanical stimuli can guide biological tissue formation, we employed a computational approach investigating the scaffold-associated mechanical and architectural properties that would enable a guided defect healing. A previous computer model of the knee joint was further developed to simulate healing of an empty osteochondral defect. Then, scaffolds were implanted in the defect and their architectures and material properties were systematically varied to identify their relevance in osteochondral defect healing. Scaffold mechanical and architectural properties were capable of influencing osteochondral defect healing. Specifically, scaffold material elastic modulus values in the range of cancellous bone (low GPa range) and a scaffold architecture that provided stability, i.e., resistance against displacement, in both the main loading direction and perpendicular to it supported the repair process. The here presented model, despite its simplifications, is regarded as a powerful tool to screen for promising properties of novel scaffold candidates fostering osteochondral defect regeneration prior to their implementation *in vivo*.

## Introduction

Articular cartilage is a connective tissue found in joints, where it enables low-friction relative movements between bones (Kheir and Shaw, 2009). As a consequence of traumas or diseases, focal chondral lesions might form in the tissue, causing pain and impairing the function of the articulation (Hunziker et al., 2015). Chondral lesions that extend also to the underlying bone are named osteochondral defects (Nukavarapu and Dorcemus, 2013).

Cartilage has no or very limited natural regenerative ability (Hunziker et al., 2015) and requires clinical intervention to enable defect healing. Moreover, if left untreated, chondral and osteochondral lesions may trigger the degeneration of the surrounding healthy tissues (Kheir and Shaw, 2009; Hunziker et al., 2015). Current clinical treatments of osteochondral defects comprise numerous surgical options. Among them can be found a tissue engineering (TE) approach of matrix-assisted autologous chondrocyte implantation (Nukavarapu and Dorcemus, 2013) and the conventional replacement-oriented strategies employing joint replacement implants. Despite the better biological potential of tissue engineering or other restorative strategies, no present clinical treatment enables a full restoration of intact articular interfaces. Some of these strategies are even associated with substantial drawbacks, such as the need for multiple surgeries (Nukavarapu and Dorcemus, 2013) or the triggering of further tissue degeneration in areas of the joint far from the original defect (Hunziker et al., 2015).

Various TE treatment strategies are presently under investigation with the aim of overcoming the limitations of current clinical treatments. The use of engineered scaffolds has been suggested for the regeneration of osteochondral defects (Nukavarapu and Dorcemus, 2013). In this context, scaffold mechanical properties might be used to induce and guide a successful tissue regeneration. In fact, the growth of bone, cartilage or fibrous tissue has been shown to be associated to different mechanical cues both *in vivo* (Claes et al., 2002) and *ex vivo* (Morgan et al., 2010), with lower strains [ <9% (Claes et al., 2002; Morgan et al., 2010)] generally found in areas of bone growth and higher strains in regions of cartilage [15–25% (Morgan et al., 2010)] and fibrocartilage [>30% (Claes et al., 2002)] formation. Moreover, several *in vitro* studies reported an influence of mechanical stimulation on mesenchymal stromal cells (MSCs) differentiation into cells of osteogenic or chondrogenic lineages (Delaine-Smith and Reilly, 2012). Specifically, lower compressive strains (10%) were shown to induce the expression of osteogenic genes in MSCs, while higher strains (15%) induced the expression of both osteogenic and chondrogenic genes (Michalopoulos et al., 2012). Despite these promising observations, the mechanical environment within osteochondral defects, and thereby the most suitable scaffold mechanical properties to foster their regeneration, are still under investigation.

Computational simulations have been developed to investigate the mechanical regulation of tissue regeneration, both in the context of bone (Prendergast et al., 1997; Claes and Heigele, 1999) and osteochondral defect healing (Duda et al., 2005; Kelly and Prendergast, 2005). For example, a mechanobiological rule for *in silico* tissue formation based on thresholds of minimum principal strain described the healing of osteochondral defects in minipigs, as verified by the comparison with histological sections (Duda et al., 2005). In another computational model, a mechanics-dependent differentiation stimulus for MSCs was calculated from octahedral shear strain and fluid velocity, again resulting in a good reproduction of the osteochondral defect repair pattern (Kelly and Prendergast, 2005). Altogether, models investigating the mechanical environment within osteochondral defects showed that the typical repair pattern, resulting in the formation of fibrous tissue at the articular interface, may be ascribed to mechanical signals, besides biological ones (Duda et al., 2005; Kelly and Prendergast, 2005).

Few computational models have aimed at investigating the effect of scaffold-supported regeneration in chondral (Koh et al., 2019) and osteochondral (Kelly and Prendergast, 2006) defects. In a model of the knee with simplified axisymmetric geometry, the optimal scaffold properties for osteochondral defect regeneration were proposed to feature a gradient from the articular surface to the base of the defect. This gradient consisted of an increasing permeability and a decreasing elastic modulus from the superficial to the deep layer of cartilage; the elastic modulus increased again in the subchondral bone region (Kelly and Prendergast, 2006). A similar configuration of scaffold properties with decreasing elastic modulus and increasing permeability from superficial to deep cartilage layers was suggested to foster the healing of chondral defects in a patient-specific 3D model of the knee (Koh et al., 2019). Importantly, both cited models assumed the whole defect to be occupied by a biomaterial, omitting the influence of scaffold architecture (Koh et al., 2019). While this approach can well represent the properties of biomaterials in the form of hydrogels, many TE strategies employ scaffolds with defined architectures (Nukavarapu and Dorcemus, 2013) that can have a non-negligible influence on strain distribution within osteochondral defects. Therefore, computational models able to evaluate the architectural properties of scaffolds, as well as their mechanical properties, have the potential of supporting further developments in the field of osteochondral TE by establishing indications for scaffold design, which could be eventually translated in improved treatment strategies.

**Figure 1:**
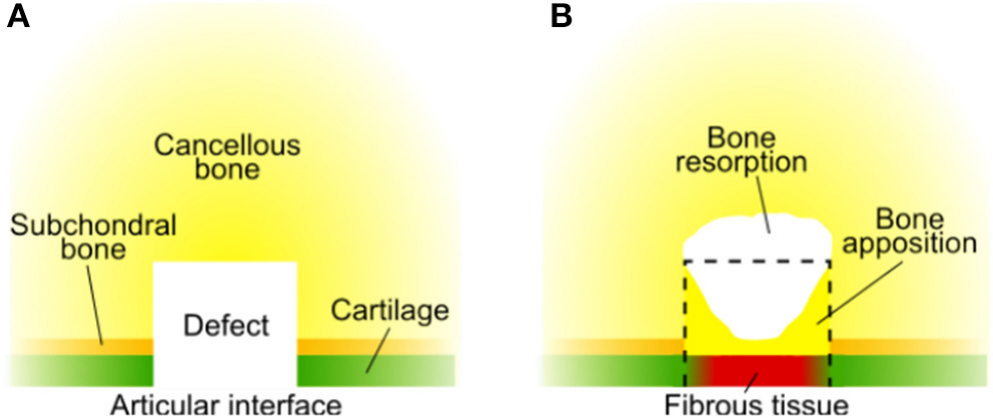
Typical repair outcome of an untreated osteochondral defect summarizing the reports from literature. **(A)** Configuration of tissues at the time of defect generation; **(B)** repair outcome. The black dashed line marks the original size of the defect. Fibrous tissue has been reported to form at the articular interface (Furukawa et al., 1980; Shapiro et al., 1993; Schlichting et al., 2008). Bone apposition at the defect sides [until the interface with cartilage (Furukawa et al., 1980; Duda et al., 2005; Lydon et al., 2019)] and bone resorption at the defect base have been reported (Jackson et al., 2001; Duda et al., 2005). Bone resorption underneath the osteochondral defect might result in the formation of cysts (Jackson et al., 2001; Schlichting et al., 2008), whose composition may vary: fibrotic tissue, fatty scar tissue, or fluid (Valderrabano et al., 2009). As numerous factors influence the repair outcome of osteochondral defects, significant differences might be observed in specific cases and this overview should be considered only as indicative.

Here, we developed a computational model to investigate the influence of scaffold mechanical properties and architecture on the healing of osteochondral defects. First, a well-established model (Kelly and Prendergast, 2005) was developed further to reproduce experimental observations of the repair of empty osteochondral defects. The repair of an empty osteochondral defect in the computational model was verified by comparison with published data. In fact, empty osteochondral defects are generally reported to form fibrous tissue or fibrocartilage as repair tissue at the articular interface (Furukawa et al., 1980; Shapiro et al., 1993; Schlichting et al., 2008) (Figure 1). Moreover, bone apposition at the sides of the defect and bone resorption at its base are often observed (Jackson et al., 2001; Duda et al., 2005), which might result in the formation of cysts (Jackson et al., 2001; Schlichting et al., 2008; Valderrabano et al., 2009).

Subsequently, the implantation of a scaffold in the defect region was modeled. The influence of scaffold properties, specifically material elastic modulus and scaffold architecture, on the repair process was evaluated. The aim was to identify suitable scaffold properties to achieve the ideal regeneration of the osteochondral defect. In this case, the ideal regeneration was defined as the development of a cartilage layer of appropriate thickness at the articular interface and the re-establishment of healthy underlying bone, without bone resorption or fibrous tissue formation.

## Materials and Methods

An iterative computer model was implemented to simulate the dynamics of the repair process within osteochondral defects. The model simulated the biological activity within the defect by using a set of equations implemented in Matlab. This model was then coupled to a finite element model, which determined the mechanical stimuli within the defect at each iteration, influencing cellular behavior. At each iteration, cellular activity (i.e., migration, proliferation, differentiation, etc.) was simulated together with the deposition of newly formed tissue, which then influenced the mechanical environment within the defect. In the following sections, a detailed description of the different components of the model is provided together with the description of the overall framework.

### Finite Element Model of the Femoral Condyle

**Figure 2:**
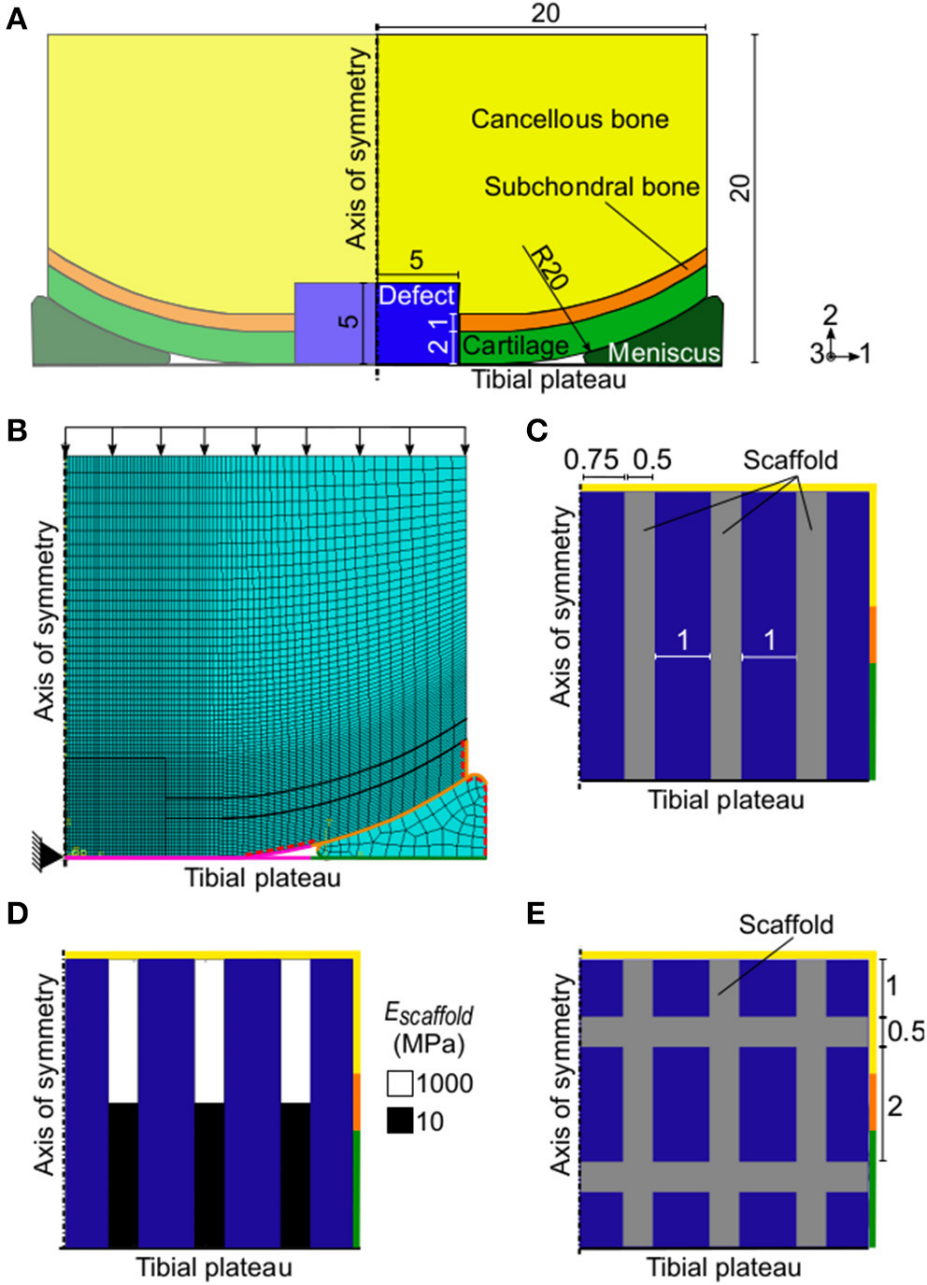
Axisymmetric FE model of a femoral condyle featuring an osteochondral defect. **(A)** FE model with empty osteochondral defect. Axes 1, 2, and 3 refer to the radial, axial, and circumferential directions, respectively; **(B)** mesh and settings of FE model. Black arrows indicate the applied pressure load. The black triangle indicates the encastre boundary condition. Magenta, orange, and green surfaces highlight the femoral condyle-tibia, cartilage-meniscus, and meniscus-tibia interactions, respectively. The red dashed lines mark the free cartilage surfaces with pore pressure of 0 MPa during the consolidation step; **(C)** and **(E)** detail of defect region with vertical struts and grid-like scaffold, respectively. The rest of the model was identical to the one with empty osteochondral defect. All reported values are in mm; **(D)** biphasic scaffold with 10–1,000 MPa elastic modulus (E_scaffold_) implemented in the defect. The colors of the borders indicate the neighboring tissues in the FE model of the femoral condyle: yellow, orange, and green stand for cancellous bone, subchondral bone, and cartilage, respectively. The blue areas represent the granulation tissue at day 1.

An axisymmetric model of a knee femoral condyle featuring cartilage, subchondral bone, cancellous bone, an osteochondral defect, a meniscus, and the tibial plateau (Figure 2A) was built in Abaqus (Dassault Systèmes, France) reproducing a previously published geometry (Kelly and Prendergast, 2005), to which in the following paragraphs will be referred as the “*Reference Model*.”

The osteochondral defect had a radius and a depth of 5 mm and it was assumed to be initially composed of granulation tissue. The size of the defect matched the one of the reference model (Kelly and Prendergast, 2005) and was within the diameter range of 3–7 mm commonly studied in *in vivo* animal models (Furukawa et al., 1980; Shapiro et al., 1993; Jackson et al., 2001; Duda et al., 2005; Schlichting et al., 2008; Ikeda et al., 2009; Lydon et al., 2019). Material properties of the tissues were defined as poroelastic (Table 1) except the healthy cartilage layer, which was modeled as hyperelastic with neo-Hookean strain energy potential and the following material parameters: *C*_10_ = 2.14 MPa and *D*_1_ = 0.399 MPa. Newly formed cartilage within the defect region was also modeled as poroelastic (Table 1), as later described. The meniscus was also poroelastic and had transversally isotropic mechanical properties, specifically: radial and axial compressive modulus of 0.5 MPa; circumferential compressive modulus of 100 MPa; axial to radial Poisson's ratio and shear modulus of 0.5 and 0.167 MPa, respectively; and Poisson's ratio and shear modulus of 0.0015 and 0.05 MPa, respectively, in the other two directions. The other material parameters of the meniscus were assigned the same values as poroelastic cartilage (Table 1). The tibial plateau was modeled as a rigid wire.

**Table 1:** Poroelastic material properties of tissues.

**Tissues**	**Elasticity (Kelly and Prendergast**, **2005****)**		**Permeability (Kelly and Prendergast**, **2005****)**		**Porous bulk moduli (Checa et al., 2011)**
	**Elastic Modulus (MPa)**	**Poisson's Ratio**	**Permeability (mm/s)**	**Void Ratio**	**Bulk Modulus of Grains (MPa)**
Cancellous bone	6,000	0.3	3.63 × 10^−8^	4	13,920
Subchondral bone	17,000	0.3	9.74 × 10^−11^	0.042	13,920
Granulation tissue	0.2	0.167	9.74 × 10^−8^	4	2,300
Poroelastic cartilage	10	0.167	4.87 × 10^−8^	4	3,700
Fibrous tissue	2	0.167	9.74 × 10^−8^	4	2,300

*The specific weight of wetting liquid in the definition of permeability was 9.74 × 10^−6^ N/mm^3^ and the bulk modulus of fluid in the definition of the porous bulk moduli was 2,300 MPa for all materials (Checa et al., 2011)*.

A finite element (FE) analysis was performed by means of a 1 s soil loading step followed by a 0.5 s consolidation step. The geometry was meshed with elements type CAX8RP (Figure 2B). Specifically, the defect region was meshed with 1,600 elements having a seed size of 0.125 mm, a mesh size which was proven to be adequate in the reference model (Kelly and Prendergast, 2005). A coarser mesh was used for the areas far away from the region of interest, with seed size reaching a maximum of 0.8 mm in the lateral and proximal sides of the femoral condyle and in the meniscus.

The interactions between femoral complex and tibial plateau (Figure 2B, magenta surfaces), cartilage and meniscus (Figure 2B, orange surfaces), and meniscus and tibial plateau (Figure 2B, green surfaces) were defined as frictionless in the tangential direction and as “hard contact” in the normal direction. Surfaces involved in the interactions were selected in such a way as to comprise also segments that would come in contact because of loading, but that were not in contact in the unloaded geometry.

A 0.637 MPa pressure load, corresponding to a 800 N force (Kelly and Prendergast, 2005), was applied on the upper surface of the cancellous bone (Figure 2B, black arrows). An encastre boundary condition was assigned to the tibial reference point, found at the axis of symmetry (Figure 2B, black triangle). Initial conditions for pore pressure and saturation were defined for the whole model with values of 0 MPa and 1 mm^3^/mm^3^, respectively. During the consolidation step, the free cartilage edges were assigned a pore pressure value of 0 MPa (Figure 2B, red dashed surfaces).

### FE Models of the Femoral Condyle Featuring a Scaffold

Axisymmetric FE models of a knee femoral condyle featuring scaffolds in the defect region were built as already described for the empty osteochondral defect, but with different defect material properties.

First, a scaffold composed of three vertical struts in the axisymmetric representation was implemented (Figure 2C). This geometry corresponded to three concentric rings in 3D. Then, a grid-like scaffold featuring both vertical and horizontal struts was investigated (Figure 2E).

All implemented scaffolds had material permeability of 3.63 x 10^−8^ mm/s, void ratio of 4, bulk modulus of grain of 0 MPa, and Poisson's ratio of 0.3. Three different material elastic moduli (*E*_*Scaffold*_) were tested: 0.1, 10, and 1,000 MPa, corresponding to an overall scaffold stiffness of 0.25, 24.5, and 2,445 N/mm, respectively, in the direction of the applied load. Moreover, a scaffold with biphasic mechanical properties was investigated, having a sharp transition of elastic modulus from 1,000 to 10 MPa in the proximal and distal regions, respectively (Figure 2D). The overall stiffness of the biphasic scaffold in the direction of the applied load was 50 N/mm. The grid-like scaffold had a material elastic modulus of 1,000 MPa and an overall stiffness of 2,573 N/mm in the direction of the applied load. All investigated cases are summarized in Table 2.

**Table 2:** List of models and corresponding investigated cases.

**Model**	**Investigated Cases**
Empty osteochondral defect	1) MSCs invasion from the bone marrow 2) Uniformly distributed MSCs
Osteochondral defect with vertical struts scaffold	1) *E_*Scaffold*_* = 0.1 MPa 2) *E_*Scaffold*_* = 10 MPa 3) *E_*Scaffold*_* = 1,000 MPa 4) Biphasic scaffold (10–1,000 MPa)
Osteochondral defect with grid-like scaffold	*E_*Scaffold*_* = 1,000 MPa

The scaffold material was chosen to have a porosity of 50%. The porosity of the scaffold material was modeled by assuming that biological tissues could occupy the percentage of scaffold struts indicated by the porosity value. Therefore, material properties of the scaffold regions at day 1 were calculated as the weighted average of scaffold properties and granulation tissue properties. The elastic modulus assigned to the scaffold struts (*E*_*Strut*_) when a material porosity *P* was implemented was given by Equation (1).

(1) $$\begin{array}{r} E_{strut}=\frac{1}{100}\left[P\cdot E_{Gran}+\left(100-P\right)\cdot E_{scaffold}\right] \end{array}$$

Where *E*_*Gran*_ and *E*_*Scaffold*_ are the elastic moduli of granulation tissue and scaffold material, respectively. Poisson's ratio, permeability, and bulk modulus of grain of the scaffold struts were similarly calculated. Material properties of the scaffold regions after day 1 were calculated as later described.

### Calculation of the Mechanical Stimulus

The mechanical stimulus (*S*) in the defect region was computed from octahedral shear strain (γ) and fluid velocity (*v*) using Equation (2) (Kelly and Prendergast, 2005).

(2) $$\begin{array}{r} S=\frac{\gamma }{a}+\frac{v}{b} \end{array}$$

Where *a* = 3.75% and *b* = 3 × 10^−3^ mm/s were empirically-derived constants (Kelly and Prendergast, 2005).

Thresholds of *S* were defined to describe the mechanics-dependent cell behavior (Table 3).

**Table 3:** Thresholds of S to describe the mechanics-dependent tissue formation (Kelly and Prendergast, 2005).

**0 ≤*S* < 0.01**	**0.01 ≤*S* < 1**	**1 ≤*S* < 3**	***S* ≥ 3**
Bone resorption	Bone formation	Cartilage formation	Fibrous tissue formation

### Model of Cellular Activities

A Matlab (MathWorks, USA) script was developed to simulate cellular activities. Four cell phenotypes were modeled: MSCs, chondrocytes, fibroblasts, and osteoblasts. The simulated cellular activities were MSCs migration, MSCs differentiation, mitosis and apoptosis.

The defect area was represented by a 40 × 40 elements matrix, in which each element corresponded to the element in the same position in the mesh of the FE model. Elements could be populated by cells of different phenotypes up to a maximum number of *N*_*MAX*_ = 100 cells/element. The value of *N*_*MAX*_ was chosen by considering that a length of 0.125 mm (corresponding to the side length of the matrix elements in the FE model) could host up to a maximum of 10 cells based on the minimum diameters of the investigated cell phenotypes: 15–30 μm for MSCs (Krueger et al., 2018); 20–50 μm for osteoblasts (Qiu et al., 2019); ~20 μm for chondrocytes (Freitas, 1999); 10–15 μm for fibroblasts (Freitas, 1999). When a scaffold was implemented, the porosity of the scaffold material enabled cells to populate the struts up to a number $N_{MAX}^{Strut}$, which depended on scaffold porosity following Equation (3).

(3) $$\begin{array}{r} N_{MAX}^{Strut}=N_{MAX}\cdot P \end{array}$$

At day 1, the defect region was empty of cells except for the elements neighboring cancellous bone, which were completely filled by MSCs. These elements were replenished with MSCs at every day, modeling a continuous cell supply from the bone marrow. This setup was used to study the healing of osteochondral defects both empty and with scaffolds and reproduced the experimental observation that all cells involved in the repair of osteochondral defects originated from marrow-derived progenitor cells (Shapiro et al., 1993). To investigate the influence of the cell source on the repair process of an empty osteochondral defect, an additional model was built featuring all defect elements filled with *N*_*MAX*_ MSCs already at day 1 (Table 2).

MSCs migration was modeled as a diffusion process. The diffusion coefficient (*D*) of each element was calculated as the weighted average of the diffusion coefficients of the tissues found in the element (Table 4), as expressed by Equation (4).

(4) $$\begin{array}{r} D=\frac{1}{N_{MAX}}\left[\left(N_{MAX}-\overset{n_{t}}{\underset{k=1}{\sum }}N_{k}\right)\cdot D_{Gran}+\overset{n_{t}}{\underset{k=1}{\sum }}D_{k}\cdot N_{k}\right] \end{array}$$

**Table 4:** Diffusion coefficients of tissues (Kelly and Prendergast, 2005) and scaffold.

**Tissue**	**Abbreviation**	**Diffusion coefficient (mm^**2**^/day)**
Granulation	*D_*Gran*_*	0.80
Cartilage	*D_*Cart*_*	0.05
Fibrous	*D_*Fibro*_*	0.10
Bone	*D_*Bone*_*	0.01
Scaffold	*D_*Scaffold*_*	0.01

Where *n*_*t*_ was the number of species and *N* was the space fraction occupied by the cells of a specific phenotype or by the scaffold (when implemented). The space fraction *N* was such as:

(5) $$\begin{array}{r} \frac{1}{N_{MAX}}\overset{n_{t}}{\underset{k=1}{\sum }}N_{k}=1. \end{array}$$

In this study, *n*_*t*_ was equal to 4 for the empty osteochondral defect, representing granulation tissue, cartilage, fibrous tissue and bone; *n*_*t*_ was equal to 5 when the scaffold was implemented. Empty space was assigned the properties of granulation tissue.

An FE model was built in Abaqus to simulate the cell diffusion process. The diffusion FE model represented only the defect area, which was meshed with the same number of elements as the main FE model (1,600 elements) with element type DC2D4. Each element was assigned diffusivity material properties as calculated with Equation (4). Moreover, the MSCs content of each element was defined as initial condition.

All cell phenotypes underwent *S*-dependent mitosis and apoptosis, as previously modeled elsewhere (Checa et al., 2011), except MSCs, whose proliferation was not *S*-dependent and had a constant 15% rate. If values of *S* in an element were in the range that would foster MSCs differentiation into a specific cell phenotype (Table 3), the already existing cells of that phenotype would perform mitosis by increasing of 5% in number. All other cell phenotypes in the element, except MSCs, would perform apoptosis by decreasing of 15% in number.

MSCs differentiation was allowed from day 1 and assumed to be completely *S*-dependent. If values of *S* in an element were in the range of a specific tissue formation (Table 3), 5% of the MSCs in the element would differentiate into cells of the specific tissue phenotype. When *S* was in the range of bone resorption, osteoblasts in the element reduced their number of 10%.

### Update of the Material Properties in the Defect

It was assumed that cells in each element would produce their corresponding tissue proportionally to their number. Therefore, properties in each element were calculated as the weighted average of individual tissue properties based on the number and phenotype of cells occupying the element. Empty space (i.e., not yet populated by cells) and space occupied by MSCs were assigned the properties of granulation tissue. Spaces occupied by chondrocytes, fibroblasts, and osteoblasts were assigned the properties of poroelastic cartilage, fibrous tissue, and bone, respectively (Table 1). When a scaffold was implemented, cells could populate *P* % of the elements belonging to the struts; the remaining space fraction of (100 – *P*) % was assigned the mechanical properties of the scaffold material.

**Figure 3:**
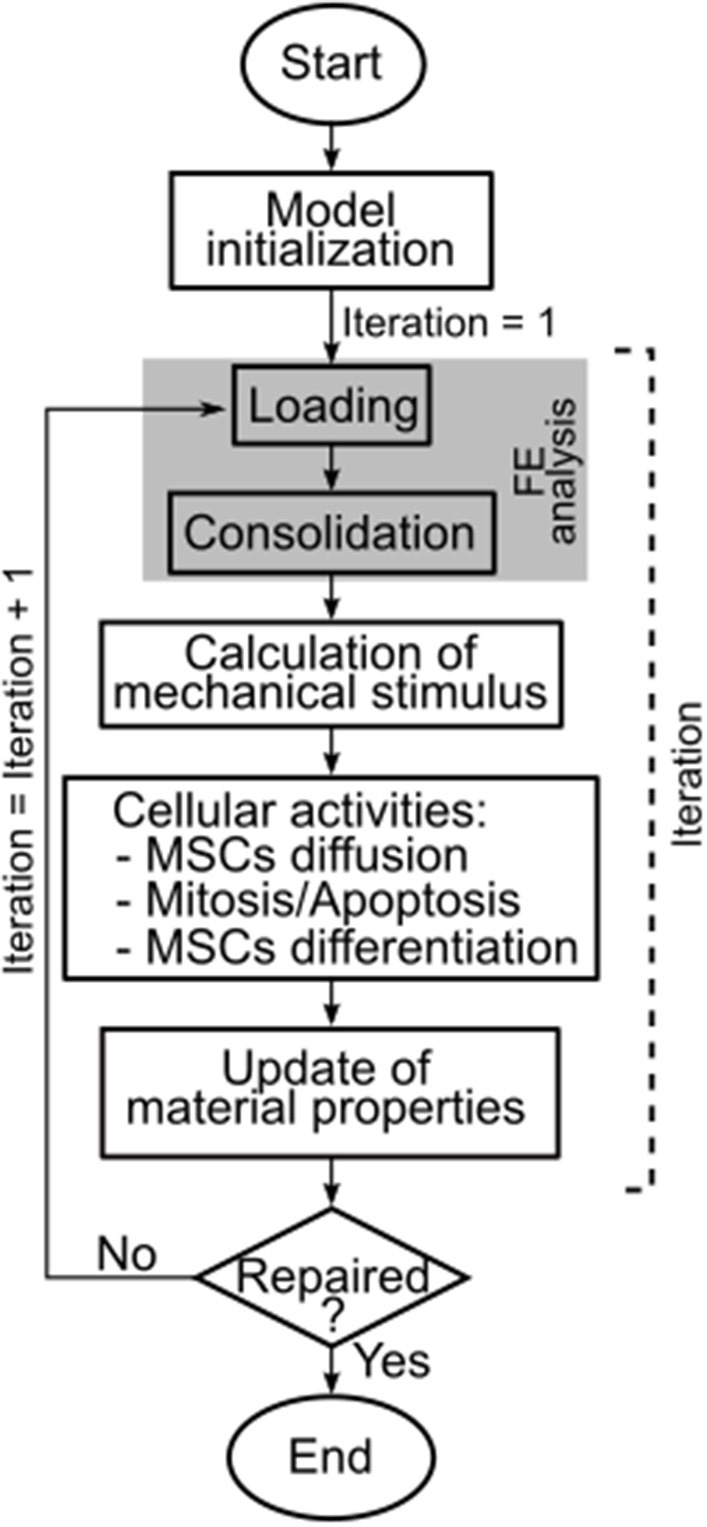
Workflow of the here presented model. The model ran until completion of the repair process, which was reached at iteration 50. FE = finite element.

Because of cellular activities, the mechanical properties of each individual element in the defect region varied throughout the simulation, i.e., throughout the repair process. Therefore, new material properties were defined for each element after the calculation of cellular activities (Figure 3). The material properties update was performed for elastic modulus, Poisson's ratio, permeability and bulk modulus of grain as the weighted average of cell number and type [see Equation (4)].

Subsequently, a new compressive FE analysis of the axisymmetric femoral condyle was performed, marking the beginning of a new iteration (Figure 3). An iteration was defined as the FE analysis of the knee femoral condyle and the calculation of the corresponding cellular activities in the defect. The model ran for 50 iterations (Kelly and Prendergast, 2005), i.e., until repair of the osteochondral defect. The completion of the repair process by iteration 50 was confirmed also for the here presented model by running it for 100 iterations and observing that the maximum difference in the amounts of formed tissues was lower than 7% of the values at iteration 50. One iteration of the model roughly corresponded to 1 day of the *in vivo* repair process.

Results were evaluated by observing the distributions of γ, *S*, and the four investigated cell phenotypes in the osteochondral defect. Importantly, at each day, cellular distributions determined the actual tissue formation predicted by the model, while the distribution of *S* indicated the tissue formation that would have been favored by the local mechanical environment.

## Results

### Repair of an Empty Osteochondral Defect With Progenitor Cell Invasion From the Bone Marrow

**Figure 4:**
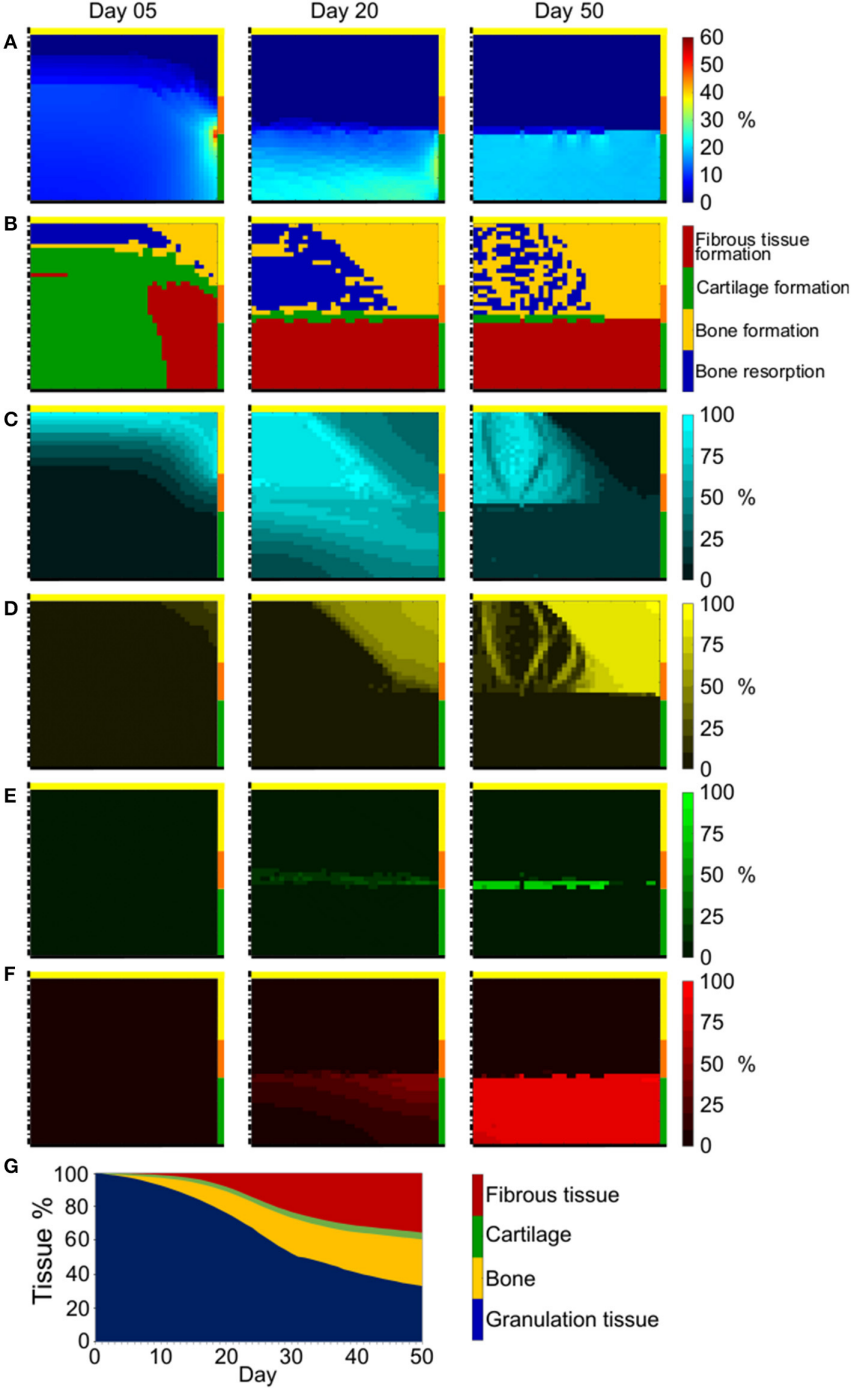
Repair outcome of empty osteochondral defect with progenitor cell invasion from bone marrow. **(A)** Distribution of γ; **(B)** prediction of tissue formation based on S; **(C–F)** amount and distribution of MSCs, osteoblasts, chondrocytes, and fibroblasts, respectively. Left, middle, and right columns show results at day 5, 20, and 50, respectively. The scale bars to interpret the plots are on the left side of the corresponding rows. The black dash-dot lines and the black solid lines mark the axis of symmetry and the articular interface, respectively. The colors of the borders indicate the neighboring tissues in the FE model of the femoral condyle: yellow, orange, and green stand for cancellous bone, subchondral bone, and cartilage, respectively; **(G)** quantification of tissues formed in the defect during the healing process.

The octahedral shear strain (γ) distribution in the empty osteochondral defect varied throughout the repair process (Figure 4A). At early time points, a high γ peak (50%) characterized the defect-cartilage-subchondral bone interface, but it smoothened and disappeared with the progression of the repair process. Lower (<1%) and higher (~20%) values of γ were found in the proximal and distal areas of the defect, respectively, with a sharp transition developing at the level of subchondral bone by the final day.

Prediction of tissue formation based on the mechanical stimulus (*S*) showed that the mechanical environment at early time points favored the formation of cartilage in the defect, with traces of fibrous tissue confined to areas neighboring the cartilage-subchondral bone interface (Figure 4B). A favorable mechanical environment for the formation of bone was predicted laterally near the cancellous bone, while an environment beneficial for bone resorption was predicted at the proximal-central base of the defect (Figure 4B). After 20 days, a region with mechanical stimulus favorable to fibrous tissue formation, with thickness comparable to the healthy cartilage, was predicted to form at the articular interface. Below this layer of fibrous tissue, the peripheral part of the defect experienced a mechanical environment that fostered the formation of bone, while a region favorable to bone resorption was predicted in the central part. This situation was maintained until the completion of the repair process (50 days), with the region favorable to bone resorption only partially substituted by a region where the mechanical stimulus promoted bone formation and a very small region favorable to cartilage formation located between fibrous tissue and an underlying bone resorption area. The prediction of tissue formation based on *S* was consistent with the implemented mechanobiological rule for tissue differentiation [Equation (2)], according to which higher values of γ would result in higher values of *S*. Growing values of *S* corresponded to a stimulus to form tissues in the following order: bone resorption < bone formation < cartilage formation < fibrous tissue formation (Table 3). Thus, areas of the defect undergoing greater straining were predicted to favor fibrous tissue formation, while regions experiencing a lower straining were indicated as favorable to bone resorption or bone formation.

Cell invasion into the defect region was limited to roughly a third of the defect area at day 5 (Figure 4C left). At this early time point, chondrocytes and fibroblasts were completely absent (Figures 4E,F left, respectively), while small amounts of osteoblasts already formed at the proximal-peripheral corner of the defect (Figure 4D left). By day 20, the cellular invasion of the defect was complete (Figures 4C–F middle) and the cellular distribution of the four modeled phenotypes matched the prediction of tissue formation based on the mechanical stimulus *S* (Figure 4B middle). Similar cell distributions were found at day 50 (Figures 4C–F right), with the additional establishment of osteoblasts bridges through the region of mixed bone resorption and bone formation (compare Figures 4B,D right).

The outcome of the repair process in the empty osteochondral defect with progenitor cell invasion from the bone marrow (Figures 4C–F right) was the development of a layer of fibrous tissue at the articular interface. Bone formed peripherally up to the level of the cartilage-subchondral bone interface of the healthy tissues. Bone resorption happened at the proximal base of the defect, generating a situation comparable to the formation of a cyst, which was partially replaced by mineralized tissue by the end of the repair process. Only a minor amount of cartilage could form in the middle region of the defect under these constrains in our model.

The quantification of tissues in the defect showed a progressive reduction of granulation tissue concomitantly to an increase of other tissue types (Figure 4G). Bone and fibrous tissue amounts steadily rose up to 27.5 and 35.2%, respectively. Cartilage did not increase as much as the other formed tissues, representing only 4.1% of the total tissue volume at the end of the repair process. After 50 days, the residual granulation tissue amount, given by the MSCs still found in the defect region, was 33.2%.

### Repair of an Empty Osteochondral Defect With Uniform Distribution of Progenitor Cells

An initial uniform distribution of MSCs in the defect region, which simulated the treatment of the osteochondral defect by means of cell therapy, resulted in minimal variations both in the distribution of γ and in prediction of tissue formation based on *S* throughout the repair process.

**Figure 5:**
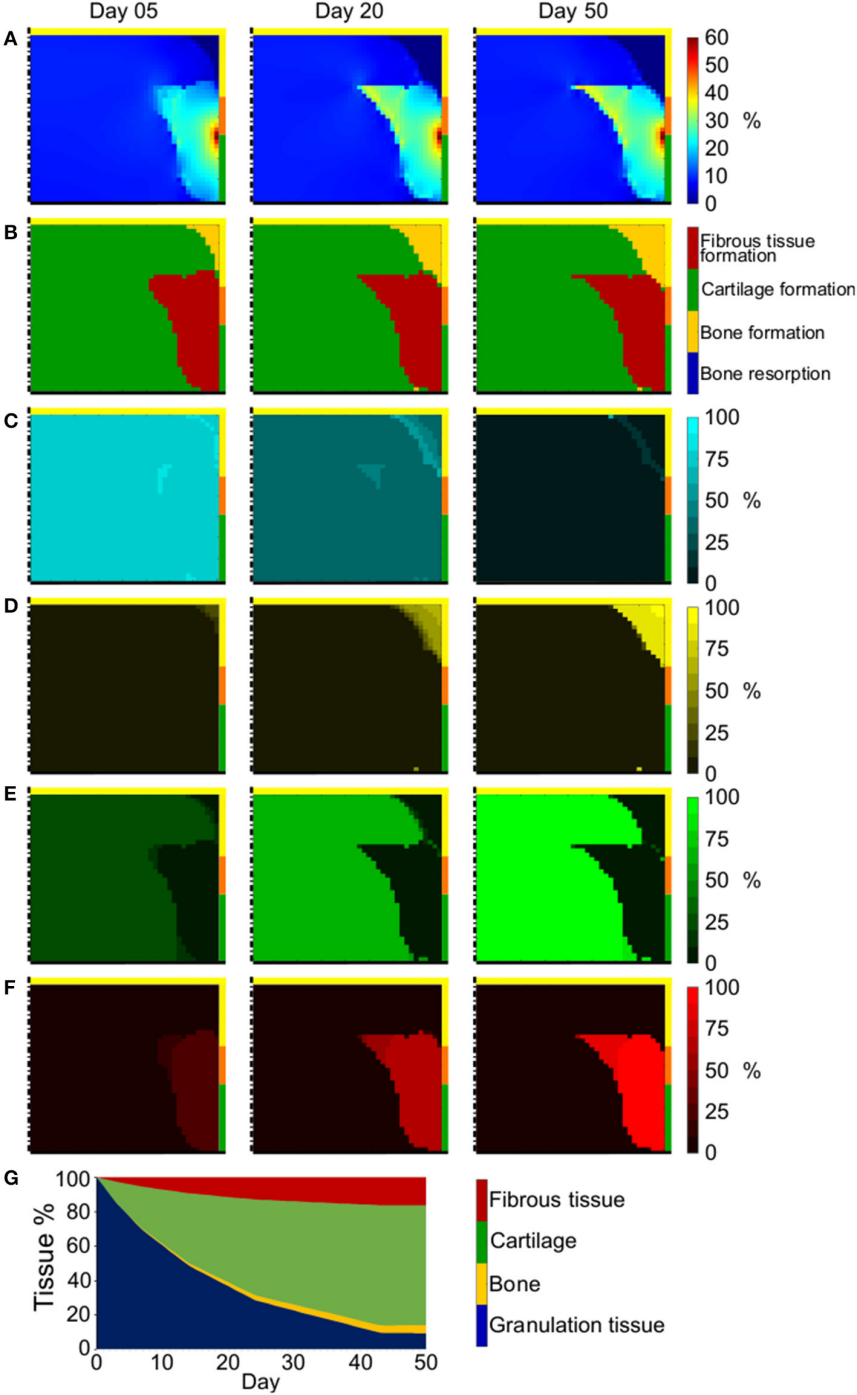
Repair outcome of empty osteochondral defect with uniformly distributed progenitor cells. **(A)** Distribution of γ; **(B)** prediction of tissue formation based on S; **(C–F)** amount and distribution of MSCs, osteoblasts, chondrocytes, and fibroblasts, respectively. Left, middle, and right columns show results at day 5, 20, and 50, respectively. The scale bars to interpret the plots are on the left side of the corresponding rows. The black dash-dot lines and the black solid lines mark the axis of symmetry and the articular interface, respectively. The colors of the borders indicate the neighboring tissues in the FE model of the femoral condyle: yellow, orange, and green stand for cancellous bone, subchondral bone, and cartilage, respectively; **(G)** quantification of tissues formed in the defect during the healing process.

At the defect-cartilage-subchondral bone interface, γ assumed high values (~20%), reaching a peak of 56% (Figure 5A). In the area between the region of high strain and cancellous bone, γ was instead very low (<5%). However, the majority of the defect experienced strain of approximately 10%. The distribution of γ was almost identical for the whole repair process, the main variation being the higher γ (up to 30%) measured at the interface between regions of moderate (~10%) and high (~20%) strain toward the end of the process.

Similarly, the prediction of tissue formation based on *S* was constant from early to late time points (Figure 5B), showing fibrous tissue formation at the defect-cartilage-subchondral bone interface as a consequence of the high values of γ found there and the applied mechanobiological rule (Equation (2) and Table 3). Bone formed at the proximal-peripheral area of the defect, in correspondence to the lowest predicted values of γ, and cartilage formed in the remaining defect region, experiencing intermediate straining.

Cellular distributions matched the prediction of tissue formation based on *S*. The number of MSCs decreased throughout the process (Figure 5C) due to their differentiation into osteoblasts in the proximal-peripheral area (Figure 5D), into fibroblasts at the defect-cartilage-subchondral bone interface (Figure 5F), and into chondrocytes in the rest of the defect (Figure 5E).

Cellular distributions at the end of the repair process were shown to exactly reproduce the prediction of tissue formation based on *S* both with progenitor cell invasion from the bone marrow and with uniform progenitor cell distribution in the defect region. Therefore, the prediction of tissue formation based on *S* at day 50 was considered as the outcome of the repair process in all subsequent analyses.

The quantification of tissue formation throughout the repair process showed a rapid reduction in granulation tissue, matched by a fast increase in cartilage (Figure 5G). The amount of fibrous tissue and bone increased more slowly and reached a lower final value. In fact, at the end of the repair process the defect region was composed of 70% cartilage, 16% fibrous tissue, 9% granulation tissue, and 5% bone.

### Repair of an Osteochondral Defect With Scaffold: Effect of Scaffold Material Elastic Modulus

A scaffold with vertical struts was implemented in the defect region and the influence of its material elastic modulus on the repair outcome was investigated. All evaluations of osteochondral defect healing with scaffolds were performed with progenitor cell invasion from the bone marrow, as this is the configuration that more closely represents the *in vivo* situation.

**Figure 6:**
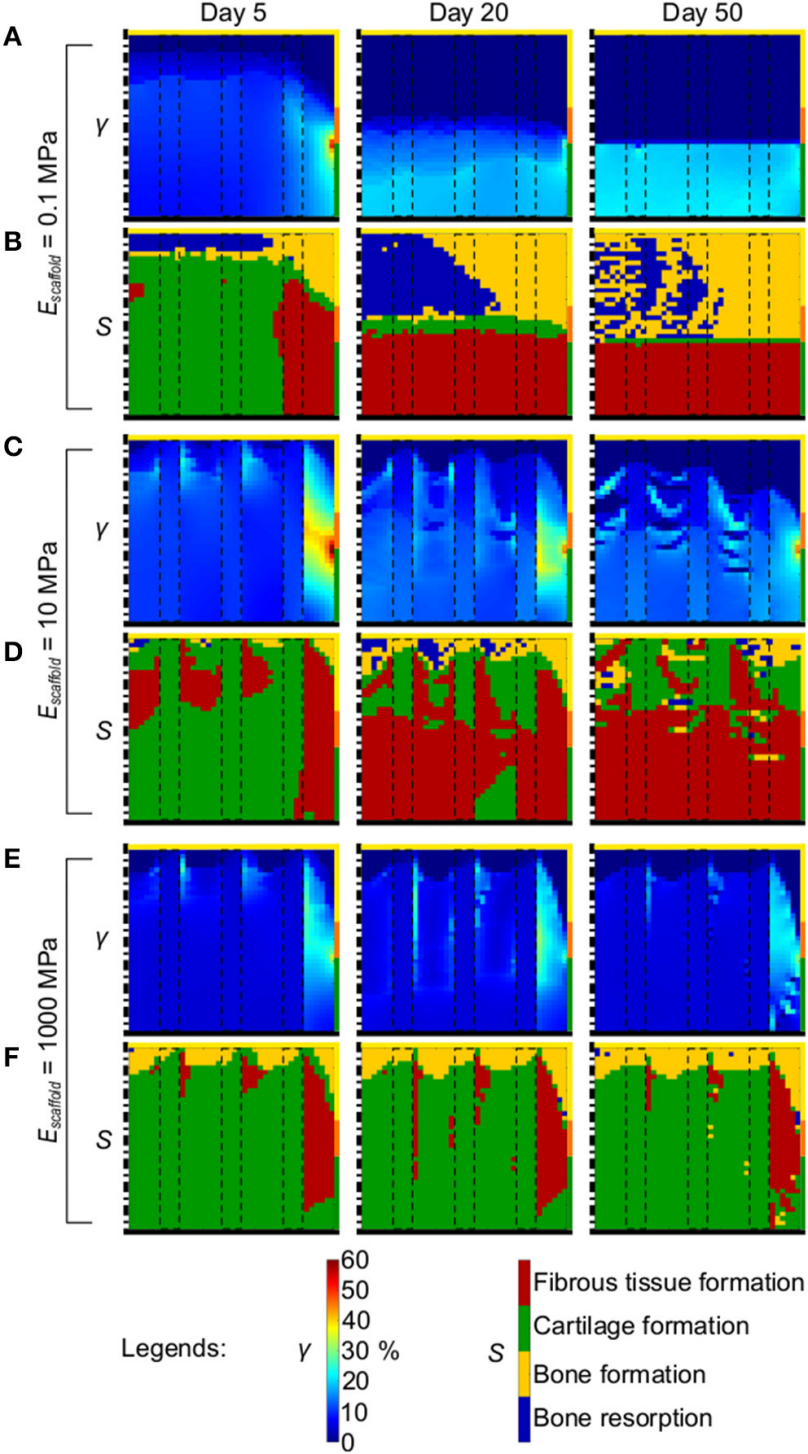
Mechanical environment in the osteochondral defect in dependency of the elastic modulus of the scaffold material. **(A,C,E)** Distribution of γ with scaffold material having 0.1, 10, and 1,000 MPa elastic modulus, respectively; **(B,D,F)** prediction of tissue formation based on S with scaffold material having 0.1, 10, and 1,000 MPa elastic modulus, respectively. Left, middle, and right columns show results at day 5, 20, and 50, respectively. The scale bars to interpret the plots are on the bottom. The black dash-dot lines and the black solid lines mark the axis of symmetry and the articular interface, respectively. The colors of the borders indicate the neighboring tissues in the FE model of the femoral condyle: yellow, orange, and green stand for cancellous bone, subchondral bone, and cartilage, respectively. The black dashed rectangles mark the struts of the scaffold.

The distribution of γ in the defect and its evolution during the repair process in presence of a scaffold with a material elastic modulus of 0.1 MPa (Figure 6A) was similar to the one of the empty defect (c.f. Figure 4A). In fact, a peak of high strain (50%) was observed at the defect-cartilage-subchondral bone interface at early time points, which subsequently disappeared. At late time points, high (~20%) and low (<1%) γ were predicted at the articular interface and in the proximal half of the defect, respectively. The prediction of tissue formation based on *S* was also comparable between empty defect (Figure 4B) and 0.1 MPa scaffold (Figure 6B), with high amounts of cartilage and fibrous tissue predicted at early and late time points, respectively, and consistently with the applied mechanobiological rule (Equation (2) and Table 3). The outcome of the repair process with the 0.1 MPa scaffold was the formation of a layer of fibrous tissue at the articular interface and bone growth from the lateral border of the defect (Figure 6B right). Moreover, bone resorption was observed in the proximal-central area of the defect and only minor amounts of cartilage formed at the interface between fibrous tissue and bone.

When a scaffold with material elastic modulus of 10 MPa was implemented in the defect, the distribution of γ at early time points showed a region of high strain at the peripheral side of the defect where γ reached 60% (Figure 6C left). Strain values in the rest of the defect were ~10%. As the repair process progressed, the distribution of γ became more irregular. At day 50, the proximal region of the defect experienced very low strain (<1%), while at the articular interface γ was 10–15% (Figure 6C right). The peripheral-lateral region maintained higher strains than the rest of the defect, with values ranging from 17 to 40%. The prediction of tissue formation based on *S* indicated that at early time points the mechanical environment would have favored the development of high amounts of cartilage and very low amounts of bone, although with no regions of bone resorption (Figure 6D left). This prediction was consistent with the distribution of γ described above. Fibrous tissue was consistently predicted to form at the peripheral side of the defect throughout the repair process, where values of γ always remained high. As time progressed, lower amounts of cartilage and higher amounts of fibrous tissue were predicted to form, corresponding to the observed increase in γ. The final outcome of the repair process with a scaffold featuring a material elastic modulus of 10 MPa was the growth of a thick layer of fibrous tissue at the articular interface and a fragmented layer of cartilage underneath it (Figure 6D right). The heterogeneous prediction of tissue formation in the proximal and middle regions of the defect matched the irregular distribution of γ found there. The growth of bone was extremely limited and confined to the proximal-lateral corner of the defect.

The implementation of a scaffold featuring a material elastic modulus of 1000 MPa resulted in low γ (<10%) in the defect throughout the repair process (Figure 6E). The proximal base of the defect experienced strain <1%, while the peripheral-lateral region reached values of γ ≈ 20%. Also the prediction of tissue formation based on *S* minimally varied with the progression of the repair process (Figure 6F). The outcome of the repair process with a scaffold featuring a comparably high material elastic modulus of 1,000 MPa revealed the growth of a thin layer of bone at the proximal base, corresponding to the region of low values of γ, and the formation of an extensive amount of cartilage in the rest of the defect experiencing intermediate values of γ (Figure 6F right). Fibrous tissue was found at the peripheral side only, where high values of γ were found. Interestingly, no bone resorption was observed.

### Repair of an Osteochondral Defect With Scaffold: Effect of Biphasic Mechanical Properties

To mimic the composition of intact osteochondral tissue, as done in a number of biomaterial approaches, a scaffold with biphasic mechanical properties was implemented in the defect and its influence on the repair process was investigated. The scaffold material featured a higher elastic modulus (1,000 MPa) in the area of desired bone formation and a lower one (10 MPa) in the region of desired cartilage formation (Figure 2D).

**Figure 7:**
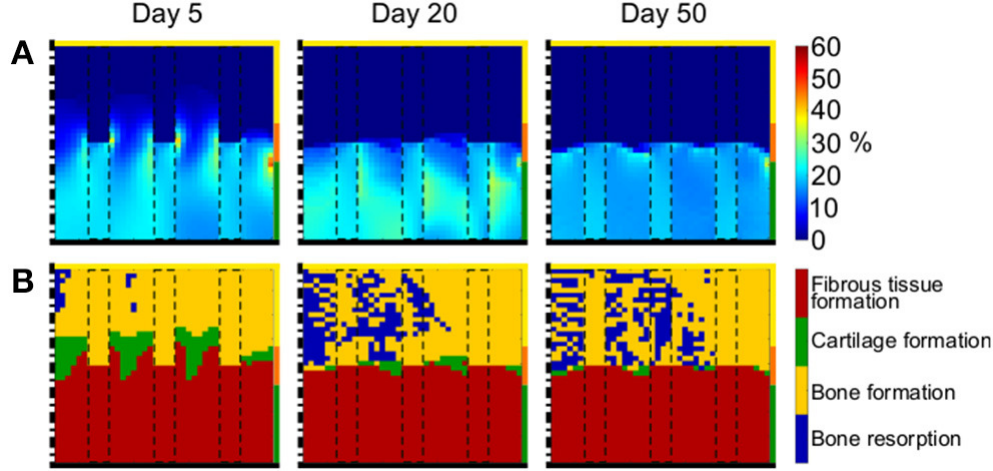
Mechanical environment in the osteochondral defect with scaffold having biphasic mechanical properties. **(A)** Distribution of γ; **(B)** prediction of tissue formation based on S. Left, middle, and right columns show results at day 5, 20, and 50, respectively. The scale bars to interpret the plots are on the left side of the corresponding rows. The black dash-dot lines and the black solid lines mark the axis of symmetry and the articular interface, respectively. The colors of the borders indicate the neighboring tissues in the FE model of the femoral condyle: yellow, orange, and green stand for cancellous bone, subchondral bone, and cartilage, respectively. The black dashed rectangles mark the struts of the scaffold.

At early time points, the distribution of γ reflected the proximal/distal difference in elastic modulus of the scaffold material (Figure 7A left). The proximal part of the defect experienced low strain (<1%), while higher strain of ~20% were measured in the distal region. Focal high peaks of γ were found at the interface between the scaffold regions with low and high elastic modulus. With the progression of the repair process, a clear distinction developed between distal and proximal half of the defect, experiencing low (<1%) and high (~20%) strain, respectively (Figure 7A middle and right).

The prediction of tissue formation based on *S* consistently indicated the formation of fibrous tissue at the articular interface (Figure 7B), in correspondence of the high values of γ. At early time points, the mechanical environment would have favored the formation of bone at the proximal base of the defect, where low values of γ were found, but vast areas of bone resorption were predicted later, indicating a further local decrease in γ. The outcome of the repair process when the biphasic scaffold was implemented was a layer of fibrous tissue at the articular interface, underneath which mixed areas of bone resorption and bone formation were found (Figure 7B right). Cartilage was present in very low quantities at the interface between bone and fibrous tissue.

### Repair of an Osteochondral Defect With Scaffold: Effect of Scaffold Architecture

The effect of scaffold architecture on the healing outcome was investigated by implementing a scaffold with a grid-like strut configuration in the defect. Additionally to the three vertical struts, the grid-like scaffold featured also two horizontal elements. Therefore, the grid-like scaffold was expected to offer resistance both against the applied compressive load and the consequent shear resulting from the radial displacement of the knee femoral condyle. The grid-like scaffold had a material elastic modulus of 1,000 MPa.

**Figure 8:**
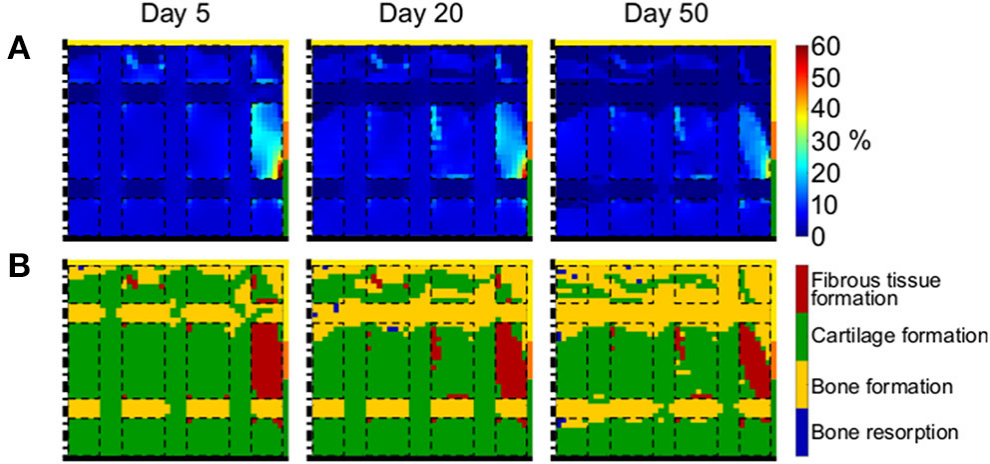
Mechanical environment of the defect region with grid-like scaffold. **(A)** Distribution of γ; **(B)** prediction of tissue formation based on S. Left, middle, and right columns show results at day 5, 20, and 50, respectively. The scale bars to interpret the plots are on the left side of the corresponding rows. The black dash-dot lines and the black solid lines mark the axis of symmetry and the articular interface, respectively. The colors of the borders indicate the neighboring tissues in the FE model of the femoral condyle: yellow, orange, and green stand for cancellous bone, subchondral bone, and cartilage, respectively. The black dashed lines mark the struts of the scaffold.

The distribution of γ varied minimally throughout the repair process (Figure 8A). The defect was generally under low strains (<5%), with the exception of the region at the subchondral bone-cartilage-defect interface, where γ ranged from 10 to 60%. Particularly low strains were measured within horizontal scaffold struts, with values lower than 1%.

The prediction of tissue formation based on *S* indicated the formation of cartilage in the whole defect, with the exception of proximal base and horizontal scaffold struts, where bone grew (Figure 8B). These results were consistent with the observed distribution of γ and indicated that the straining within horizontal struts was low enough to support bone formation, but not to elicit bone resorption. In fact, no areas of bone resorption were predicted at any time point. Moreover, low amounts of fibrous tissue were predicted to form at the subchondral bone-cartilage-defect interface, where high values of γ were found.

## Discussion

Scaffold-based strategies have a great potential for the treatment of osteochondral defects, however the design of those scaffolds remains a challenge. In this study, an *in silico* model was developed with the aim of analyzing the mechanics-dependent repair process of an osteochondral defect and the influence of scaffold mechanical and architectural properties. We show that architecture and material mechanical properties have a great influence on the mechanical signals at the defect site during the healing process and, consequently, on the healing outcome. In addition, our results suggest that scaffolds with material stiffness in the range of cancellous bone and an architecture stable against both compression and shear might foster the mechanics-dependent healing of osteochondral defects, representing a promising strategy for new clinical treatments.

When studying the repair process of an empty osteochondral defect with the here proposed adapted model, cellular activities were found to profoundly influence the mechanical environment in the defect region. In fact, at early time points, cells had not extensively populated the defect yet (Figures 4C–F left). Therefore, the mechanical input, favoring the growth of large amounts of cartilage (Figure 4B left), was not translated into tissue formation by MSCs differentiation. The invasion of MSCs into the defect region proceeded from proximal to distal and from peripheral to central areas, accordingly to the cell source [i.e., the bone marrow within cancellous bone (Shapiro et al., 1993)]. Thus, MSCs would first experience mechanical stimuli within the bone resorption, bone formation, and fibrous tissue formation thresholds, causing their differentiation into osteoblasts in the proximal-peripheral corner of the defect and into fibroblasts at the distal-peripheral side. This seemed to trigger a positive feedback loop, where the development of bone and fibrous tissue created a mechanical environment fostering their further formation (Figure 4B middle). At this point, cells had fully populated the defect and MSCs could respond to the differentiation stimulus, turning into osteoblasts and fibroblasts (Figures 4D,F middle). The suggested positive feedback loop was supported by the quantification of tissue formation, which revealed a steady increase of bone and fibrous tissue throughout the process (Figure 4G), and by the repair outcome of the empty osteochondral defect uniformly populated by MSCs from the first day (Figure 5B right).

In fact, when MSCs were initially seeded within the whole defect region, MSCs could uniformly respond to the differentiation stimulus already at early time points, a situation resulting in the filling of the defect with cartilage, with traces of bone in the proximal-peripheral region and fibrous tissue forming at the interface with native cartilage. These observations suggest that the typical repair outcome of an osteochondral defect might strongly depend on both the mechanical stimuli and the initial spatial location of progenitor cells. These results indicate that a treatment strategy able to supply uniformly distributed MSCs might result in improved defect healing, provided that the MSCs remain viable throughout the repair process. However, it is important to notice that, despite the higher formation of cartilage, the healing outcome fostered by a uniform distribution of MSCs did not re-establish the native tissue composition, as predicted by the model. In fact, a healthy subchondral bone region was not formed. Moreover, fibrous tissue grew at the interface with the native cartilage, indicating that the integration of repair and healthy tissues might be a critical point for such a potential treatment strategy. The use of MSCs to treat chondral and osteochondral defects has already been suggested in a wide variety of techniques, both with and without biomaterial scaffolds (Bornes et al., 2014; Murata et al., 2020a). Clinical studies reported complete defect filling in 70% (Buda et al., 2010), 45% (Giannini et al., 2013), and 65% (Kyriakidis et al., 2020) of patients treated with MSCs implantation in combination with a hyaluronic acid scaffold. However, an intact subchondral bone layer was re-established only in 30% (Buda et al., 2010), 35% (Giannini et al., 2013), and 10% (Kyriakidis et al., 2020) of the patients, respectively, supporting the observations derived from our computational model. In a preclinical study in rabbits, MSCs implantation without a support scaffold showed the formation of cartilaginous tissue few months after implantation concomitantly to inadequate subchondral bone formation, similar to the prediction of our model (Murata et al., 2020b). In addition, the formation of a smooth cartilaginous tissue in osteochondral defects treated with 3D MSCs constructs was observed also in pig (Murata et al., 2018). The implantation of MSCs-seeded tri-layered alginate/poly(lactic-co-glycolic) acid (PLGA) scaffolds in osteochondral defects in rabbit resulted in the formation of cartilage-like tissue (Reyes et al., 2012), as in our model. However, the same study reported a complete repair of the subchondral bone layer and a good integration between repair and healthy cartilage in some specimens (Reyes et al., 2012), contrary to our prediction. In this case, MSCs were implanted in the osteochondral defects in combination with a biomaterial, which might have influenced cellular behavior and thereby the repair outcome.

Despite the results obtained with the uniformly distributed MSCs, this work focuses on the influence of scaffold mechanical and architectural cues on osteochondral defect healing. Therefore, the incorporation of additional biological stimuli in the scaffolds, e.g., pre-seeding with MSCs, which would also result in more demanding regulatory requirements for the clinical translation process, was not investigated here and all the simulations in which scaffolds were implemented were performed with progenitor cell invasion from the bone marrow, mimicking the *in vivo* situation (Shapiro et al., 1993). For this reason, the healing outcome of the empty osteochondral defect with progenitor cell invasion from the bone marrow will be discussed more in depth in the following paragraphs.

The repair process observed in the here presented model showed strong similarities with the reference model (Kelly and Prendergast, 2005). For example, the defect area could be divided in both cases into three regions of tissue formation: a superficial, middle and deep zone, where fibrous tissue, cartilage and bone formed, respectively (Figure 4B). These observations were consistent with those reported for the healing of osteochondral defects in rabbit (Shapiro et al., 1993). However, significant differences between the here presented and the reference model were also observed, as it could be expected from the implementation of different algorithms describing cellular behaviors. Here, the deep zone of the osteochondral defect could be further divided into a peripheral area of bone formation and central area of bone resorption (Figure 4B), similarly to what was observed in the healing of osteochondral defects in minipig (Duda et al., 2005) and in goat (Jackson et al., 2001). However, bone formed both at the side and at the base of osteochondral defects in sheep (Lydon et al., 2019), suggesting a certain degree of inter-species variability in healing pattern.

In the reference model (Kelly and Prendergast, 2005), significantly different amounts of tissues formed in the defect region throughout the repair process compared to the here reported study: higher quantities of bone (~65%) and cartilage (~20%) and lower quantities of fibrous tissue (~15%) and granulation tissue (0%) were found in the defect by the end of the simulation. These differences could mostly derive from the mechanics-dependent mitosis/apoptosis behavior here implemented and from the fact that here only MSCs migrated. In this study, the rates of cell mitosis/apoptosis and MSCs differentiation were established by a sensitivity analysis of the model (Supplementary Material 1). The resulting values were comparable to previously published literature for the apoptosis rate, which in other works ranged from 5 to 16% (Checa et al., 2011), but they were lower for both mitosis, spanning from 20 to 60% in previous work (Checa et al., 2011), and MSCs differentiation, which was set to be 30% elsewhere (Checa et al., 2011). Trends in tissue formation similar to those here observed were reported for osteochondral defects both *in vivo* (Duda et al., 2005; Schlichting et al., 2008) and *in silico* (Koh et al., 2019), with the repair process resulting in higher amounts of bone and fibrous tissue than cartilage. However, a precise comparison of tissue quantification is difficult due to differences in sizes and locations of the osteochondral defects, as well as in the types of models investigated. Additionally, significant individual differences in the *in vivo* healing were reported even for subjects equally treated within the same study (Furukawa et al., 1980). Thus, model verification will rely mostly on the analysis of the types and distributions of the formed tissues, rather than on matching their quantities. The formation of bone up to the level of the cartilage interface during the healing of empty osteochondral defects was reported *in vivo* (Furukawa et al., 1980; Duda et al., 2005; Lydon et al., 2019) and *in silico* (Duda et al., 2005), consistently with our results (Figures 4B,D right). Moreover, *in vivo* tests (Furukawa et al., 1980; Schlichting et al., 2008) reported fibrous tissue or fibrocartilage to be the repair tissue at the articular interface, similarly to the prediction of the here presented model (Figures 4B,F right). The formation of cysts below empty osteochondral defects, here observed in Figure 4B, was also reported in goat (Jackson et al., 2001), in sheep (Schlichting et al., 2008) and in clinical practice (Valderrabano et al., 2009).

Altogether, the comparison of the repair process of an empty osteochondral defect between the here presented model and previous reports indicated that the model reproduced the natural repair process with good approximation, despite *in vivo* variability and model simplifications. Such simplifications included the simplified geometry and loading scenario of the knee joint, in terms of both the type of applied load (i.e., pure compression) and its low magnitude compared to the peak axial forces of 3,372–4,218 N that were measured with instrumented implants in knee replacements (Bergmann et al., 2014). In addition, the tibial plateau was modeled as non-deformable, while in the physical knee joint the femoral condyle contacts the deformable tibial cartilage. However, a test model featuring a cartilaginous tibial plateau showed no significant alterations of the repair process compared to the model with the non-deformable tibial plateau (Supplementary Material 2). Other simplifications were the passive movement of MSCs by diffusion as opposed to active cellular migration and the assumed direct correlation between cell number and formation of the corresponding tissue. For example, the value assigned to the maximum number of cells per element was an approximation, as it did not take into account the physical space occupied by extracellular matrix nor differences in tissue cellularity.

Subsequently, the verified model of osteochondral defect repair was used to study the influence of scaffold implantation on the healing process. It has been previously shown *in vivo* that scaffolds implanted without cell pre-seeding might influence the healing of osteochondral defects based on their mechanical and architectural properties (Schlichting et al., 2008; Ikeda et al., 2009). Moreover, *in silico* models to assess the influence of scaffold properties on the healing outcome of osteochondral defects have been previously published both with simplified axisymmetric (Kelly and Prendergast, 2006) and patient-specific 3D (Koh et al., 2019) geometries. In neither of the cited computational studies, however, the physical shape of the scaffold was taken into account. Such an approach may be suitable to model hydrogel scaffolds. However, numerous TE approaches employ scaffolds whose structure is not isotropic and thereby not negligible. Such structures generate a non-uniform mechanical environment and might influence cell accessibility to different regions of the defect. Here, scaffolds with a simple but distinct internal architecture were modeled in the osteochondral defect, introducing a novel approach compared to previous studies.

Initially, a scaffold composed of three vertical struts, which are the axisymmetric representation of three concentric rings, was implemented in the defect region (Figure 2C). The choice of geometry was motivated by the observation that load transmission between articular interface and defect base by means of the vertical struts might have prevented the previously observed bone resorption. The influence of the elastic modulus of the scaffold material on the repair outcome was investigated by studying three elastic moduli (0.1, 10, and 1,000 MPa).

The mechanical properties of the low stiffness scaffold with 0.1 MPa material elastic modulus matched the order of magnitude of certain biological tissues, such as granulation tissue (Table 1), as well as of macroporous scaffolds from synthetic polymers (Ikeda et al., 2009). An even lower stiffness has been reported for macroporous scaffolds from natural polymers, whose value could reach the low kPa range (Petersen et al., 2018). When the 0.1 MPa scaffold was implemented, the mechanical environment within the defect (Figures 6A,B), and thereby the mechanics-dependent repair process, was analogous to that of the empty osteochondral defect (Figures 4A,B). This low-stiffness scaffold failed in avoiding bone resorption in the central-proximal region of the defect and in preventing the formation of fibrous tissue at the articular interface, as the ability of the scaffold material to transmit mechanical loads to the base of the defect was insufficient.

Some biological soft tissues, such as cartilage and fibrous tissue, have elastic modulus in the low MPa range (Table 1), similarly to the medium stiffness scaffold featuring a material elastic modulus of 10 MPa. Moreover, TE scaffolds from biodegradable polymers can have stiffness in the order of tens of MPa (Di Luca et al., 2016) or slightly higher (Schlichting et al., 2008; Di Luca et al., 2016). As observed here, a scaffold with a material elastic modulus of 10 MPa inhibited the growth of a continuous subchondral bone layer and, although it promoted the formation of cartilage at the proximal base of the defect, it fostered the formation of a fibrous tissue layer at the articular interface that was thicker than the one observed in the empty osteochondral defect (Figure 6D right).

The high stiffness scaffold featured a material elastic modulus of 1,000 MPa, which was comparable to cancellous bone [100–2,000 MPa (Bose et al., 2012), although values up to 10–15 GPa were reported for the trabecular bone material depending on the measurement method (Rho et al., 1993)]. The implementation of the high stiffness scaffold resulted in the growth of a thin layer of bone at the proximal base of the defect (Figure 6F right). The majority of the repair tissue consisted of cartilage, with fibrous tissue forming at the interface with the neighboring healthy tissues.

Our observations indicated that scaffolds might exert a great influence on the repair process of an osteochondral defect depending on their mechanical properties. Specifically, scaffolds whose material is too soft (kPa range) might not influence the mechanics-dependent healing outcome at all, as they would not significantly alter the mechanical environment of the defect. Moreover, we observed that using scaffolds with elastic modulus matching that of cartilage (10 MPa) might not yield the desired healing outcome, but suppress the regeneration of the subchondral bone layer while favoring the formation of fibrous tissue at the articular interface. Amongst the investigated scaffold properties, the most promising healing outcome was achieved with a scaffold whose mechanical properties matched those of cancellous bone (low GPa range). When this scaffold was implemented, bone resorption at the proximal base of the defect was avoided and cartilage formed at the articular interface. However, the healing was not ideal, as subchondral bone did not grow until the healthy bone-cartilage interface and fibrous tissue formed at the peripheral side of the defect, suggesting difficulties in the integration between repair and healthy cartilage. Stiff polylactide-co-glycolide (PLGA) scaffolds were found to support the formation of more subchondral bone compared to soft PLGA scaffolds *in vivo* (Schlichting et al., 2008), similarly to our results. On the other hand, no influence of scaffold stiffness on cartilage formation was observed in the cited study. However, the reported difference in elastic modulus between stiff and soft PLGA scaffolds, i.e., 150 and 95 MPa (Schlichting et al., 2008), respectively, was lower than the one investigated here. Moreover, the architectures of stiff and soft scaffolds were different, making it difficult to ascribe the observed variations in healing purely to mechanics. Three PLGA scaffolds with elastic modulus ranging from 0.0047 to 0.26 MPa were found to result in the formation of fibrous tissue at the articular interface at the third week post implantation *in vivo* (Ikeda et al., 2009), supporting our observations. At later time points, the scaffolds with the lower stiffness resulted in better defect filling. However, also in this case, the investigated scaffolds varied not only in stiffness, but also in architecture, having porosity ranging from 80 to 95%. Thus, the better healing outcome obtained with the softer scaffolds was ascribed to their higher porosity, rather than to the influence of scaffold-dependent mechanical cues.

Despite the encouraging results obtained with the high stiffness scaffold, a concern in implanting such a scaffold is that its struts might impinge on the opposing articulating cartilage of the joint, triggering cartilage degeneration (in this case on tibial side). This might be the case even if the surface properties of the scaffolds are optimized, e.g., by rounding edges and/or by locally modifying the architecture to increase the contact area between scaffold material and opposing cartilage. Therefore, a scaffold with biphasic mechanical properties was evaluated, maintaining the favorable high elastic modulus (1,000 MPa) in the proximal half and matching the cartilage elastic modulus (10 MPa) in the distal half, i.e., at the articular interface (Figure 2D). Gradients of properties in scaffolds have already been suggested for improved healing of osteochondral defects in numerous experimental (Nukavarapu and Dorcemus, 2013; Longley et al., 2018) and computational (Kelly and Prendergast, 2006; Koh et al., 2019) studies. Specifically, scaffolds with biphasic properties have been extensively investigated with the aim of improving both bone and cartilage healing (Longley et al., 2018). Interestingly, our results indicate that implementing a biphasic scaffold with higher stiffness in the region of desired bone formation generates a mechanical environment analogous to that found in an empty osteochondral defect, especially at late time points (compare Figure 7A to Figure 4A). In fact, in both cases, tissues experienced low and high strain in the proximal and distal regions, respectively. Consequently, the mechanics-dependent repair outcome was also comparable, with bone and fibrous tissue forming in the proximal and distal regions, respectively (compare Figure 7B to Figure 4B). Compared to the scaffolds with uniform material elastic modulus of 10 and 1,000 MPa, the biphasic scaffold resulted in more bone formation (compare Figures 6D,E to Figure 7B). However, the formation of cartilage at the articular interface was not observed with the biphasic scaffold, as opposed to the high stiffness single phase scaffold (compare Figure 6E to Figure 7B). Altogether, our observations suggest that scaffolds with matching mechanical properties to bone and cartilage in the proximal and distal regions, respectively, might not be of advantage in fostering the ideal osteochondral defect healing by means of mechanical cues. However, a verification of these results by comparison with literature is particularly challenging, as biphasic scaffolds are mostly produced by combining two (or more) different materials (Nukavarapu and Dorcemus, 2013; Longley et al., 2018). Thus, the influence on healing resulting from different scaffold mechanical properties in the bone and cartilage regions cannot be distinguished from the influence resulting from different scaffold architecture and chemistry.

Finally, a grid-like scaffold featuring both vertical and horizontal elements and a material elastic modulus of 1,000 MPa was implemented (Figure 2E). The aim was to stabilize the structure against the radial displacement by means of the horizontal struts, thereby reducing the shear strain and fostering the formation of bone. Indeed, strains within horizontal struts were extremely low (Figure 8A) and bone was consistently predicted to form there throughout the repair process (Figure 8B). As a result, more bone formed when the grid-like scaffold was implemented compared to the vertical struts scaffold with the same material elastic modulus (compare Figure 8B to Figure 6F). At the same time, cartilage formation at the articular interface was not impaired and lower amounts of fibrous tissue were observed with the implementation of the grid-like scaffold. Importantly, the ideal osteochondral defect healing was not achieved with the grid-like scaffold either, as a healthy subchondral bone layer was not fully restored and bone formed ectopically within the distal horizontal strut. This outcome is regarded to be sub-optimal, as the high stiffness of the ectopic ossification might have detrimental effects on the surrounding and articulating cartilage during joint movements. Moreover, the investigated grid-like scaffold presented the risk of impinging on the articulating cartilage due to the high elastic modulus of its material, similarly to the scaffold with three vertical struts discussed above. However, our results indicate that scaffold architecture, together with scaffold stiffness, can play a key role in steering the mechanics-dependent healing of osteochondral defects. In future, scaffold development might aim at optimizing the combination of mechanical and architectural properties to achieve the ideal healing of osteochondral defects. For example, the here observed issue of ectopic bone formation might be addressed by modifying the vertical positions of the horizontal struts to allow bone formation in the desired regions and, at the same time, guarantee enough stability against the lateral displacement also at the articular interface. Additionally, the results of the simulation indicate that a material stiffness in the range of cancellous bone (low GPa range) could be of advantage. This knowledge could be used in the design of scaffold fulfilling target properties, for which various methods have already been suggested and applied. One of such methods is topology optimization, which has been used, for instance, to design scaffolds for bone tissue engineering with optimized stiffness and porosity (Challis et al., 2010; Kang et al., 2010). In another case, a combination of computer-aided design, design of experiment, and finite element analysis has been employed to define the architecture of scaffolds for bone tissue engineering in compliance with mechanical and porosity requirements (Marchiori et al., 2019).

The *in silico* model here implemented presented some limitations. First, the axisymmetric scaffold architectures here studied were simplified compared to the 3D scaffold morphologies currently investigated experimentally. Thus, our findings cannot be directly translated into the production of scaffolds with optimized properties for osteochondral defect healing, but can only be used as indications. Moreover, only two simplistic scaffold architectures were investigated, which by no means exhausted the possible range of architectural parameters that could be varied, e.g., strut thickness, strut number, strut orientation, distance between struts, and so forth. The architectural features, particularly the pore size, have been shown to play a primary role in determining the compressive modulus of scaffolds (Marchiori et al., 2019). Thus, the analysis of scaffolds with systematic variations of architectural features might result in designs supporting an improved osteochondral defect healing compared to the ones reported here. Second, cellular migration was modeled as a passive diffusion process, which precluded the evaluation of the cell-scaffold interactions that could have been expected during active cellular migration. In fact, numerous cell phenotypes, amongst which MSCs and fibroblasts, have been shown to adapt their migration behavior *in vitro* based on substrate stiffness (Pelham and Wang, 1997; Lo et al., 2000), topography (Berry et al., 2004; Park et al., 2009; Werner et al., 2017) and porosity (Harley et al., 2008; Chang et al., 2016). Third, the model did not allow studying the active remodeling process of the tissues surrounding the defect. Despite the aforementioned limitations, important insights were provided on osteochondral defect healing in presence of scaffolds. Moreover, the here presented model offers a platform to evaluate conditions that are difficult to achieve experimentally, such as a clear separation of influences derived from scaffold architectural and mechanical properties (Schlichting et al., 2008).

In conclusion, an *in silico* model of osteochondral defect healing was successfully developed to study the influence of scaffold mechanical and architectural properties, as well as of progenitor cell source, on the outcome of the repair process. Our findings indicate that the typical repair outcome of empty osteochondral defects depends on the modality of progenitor cell invasion into the defect in combination with their differentiation response to mechanical stimuli. The material stiffness and architecture of the scaffold enable the definition of mechanical cues. This quality may be used to steer the healing process and consequently the healing outcome. Specifically, scaffolds with stiffness properties comparable to cancellous bone (low GPa range) and with an architecture stable against both compression and shear fostered an improved repair outcome. However, the ideal or complete healing of the osteochondral defect could not be realized in any of the investigated cases. Moreover, low material stiffness values did not seem to support osteochondral defect healing. Such analyses can serve as basis for the design of scaffolds for improved osteochondral defect healing, specifically in respect to their mechanical and architectural properties.

The raw data, the computer models and the code supporting the conclusions of this article will be made available by the authors, without undue reservation, to any qualified researcher.

## Data Availability Statement

The raw data supporting the conclusions of this article will be made available by the authors, without undue reservation.

## Author Contributions

SC and MT designed the study. MT and KE developed the *in silico* models and collected the data. MT, SC, AP, and GD interpreted the data. MT and SC drafted the manuscript. All authors read and revised the manuscript and approved its content.

## Conflict of Interest

The authors declare that the research was conducted in the absence of any commercial or financial relationships that could be construed as a potential conflict of interest.
