# Supplementary material for: Scaffold-Dependent Mechanical and Architectural Cues Guide Osteochondral Defect Healing in silico
Source: Front Bioeng Biotechnol. 2021 Feb 15;9:642217. doi: 10.3389/fbioe.2021.642217 (PMC7917217; doi:10.3389/fbioe.2021.642217)
Supplement: Supplementary file 1 [file Table_1.docx]

Supplementary Material

# Sensitivity analysis

When calculating cellular activities, different values of maximum cell number per element, MSCs differentiation rate, mitosis and apoptosis were tested and their influence on the outcome of the repair process was evaluated before selecting a set of values to be used in all the reported simulations. In this section, each tested condition is given a code name: letters indicate a specific parameter and the following numbers indicate the imposed value, as listed in Supplementary Table 1. Coding letters that are not included in the name of a set of tested conditions were assigned a constant value, which is specified in the following paragraphs.

**Supplementary Table 1.** Parameters investigated in sensitivity analysis.

| **Parameter** | **Abbreviation** | **Investigated values** |
| --- | --- | --- |
| Maximum cell number per element (cells/element) | N | 49  100  144 |
| MSCs differentiation rate (%) | D | 5  10  30  50 |
| Mitosis rate (%) | M | 5  15 |
| MSCs mitosis rate (%) | MSC | 5  15 |
| Apoptosis rate (%) | A | 5  15 |

First, a model without mechanics-dependent apoptosis (except for fibroblasts) and a 5% mitosis rate was implemented, following the reference model (Kelly & Prendergast, 2005). In this model configuration, the influence of MSCs differentiation rate (values of 10%, 30%, and 50%) and maximum cell number per element (values of 49, 100, and 144) were evaluated. The investigated MSCs differentiation rates were chosen because a value of 30% was previously employed in computational modelling (Checa, Prendergast, & Duda, 2011); then, a higher and a lower value were selected (±20%). The values for the maximum cell number per element were chosen based on the size of the elements (0.125 x 0.125 mm) and the minimum diameters of the investigated cell phenotypes (10-20 µm, see section “Model of Cellular Activities”). It was assumed that a maximum of 10 x 10 cells could be found in the elements; then, a higher (12 x 12 cells) and a lower (7 x 7 cells) value were investigated.

By varying the MSCs differentiation rate with constant maximum cell number per element (N=100 cells/element), it was observed that higher differentiation rate values resulted in a slower cellular invasion of the defect (see groups N100D10, N100D30, and N100D50 in Supplementary Figure 1A). At the same time, the percentages of the formed tissues at day 50 were comparable for the three groups, indicating that higher values of MSCs differentiation fostered a faster tissue formation in the areas of the defect populated by cells. Variations of maximum cell number per element with equal MSCs differentiation rate (10%) did not considerably influence the process of cellular invasion of the defect (see groups N100D10, N49D10, and N144D10 in Supplementary Figure 1A). However, they influenced the speed of tissue formation, as more or less cells were available to the differentiation process. All the investigated cases resulted in a similar prediction of tissue formation based on the mechanical stimulus at day 50 (Supplementary Figure 1C), specifically: fibrous tissue formation at the articular interface, bone formation at the proximal-peripheral corner of the defect, bone resorption in the proximal-central and middle areas, and almost complete absence of cartilage formation.

It was observed that the prediction of tissue formation based on the mechanical stimulus and the tissue formation given by the cellular distributions did not match at the end of the repair process (compare Supplementary Figure 1C and D). Moreover, cellular phenotypes were found in areas of the defect consistently indicated as unfavourable to the formation of the corresponding tissue over numerous consecutive days, reflecting the “mechanical history” of the defect rather than responding to the concurrent mechanical environment, as expected in the physiological situation. Therefore, a mechanics-dependent apoptosis was introduced, as previously reported in literature (Checa et al., 2011). A maximum number of 100 cells/element was chosen to be used in all the subsequent simulations, as it was deemed appropriate for the evaluated sizes, while 144 cells/element would be a too high number for the employed elements, and it did not greatly reduced the speed of tissue formation, as opposed to the value of 49 cells/elements. An initial value of 10% was selected for the MSCs differentiation rate, as it resulted in the fastest cellular invasion of the defect.

When the model with mechanics-dependent apoptosis was implemented, different values of mitosis rate (5% and 15%), apoptosis rate (5% and 15%), and MSCS differentiation rates (5% and 10%) were tested. Moreover, the mitosis rate of MSCs was varied independently, as a faster mitosis was employed for stem cells than for the other cell phenotypes in literature (Checa et al., 2011). The values of mitosis rate were chosen by employing the value of 5% reported in the reference model (Kelly & Prendergast, 2005) and a higher one (+10%). The chosen values for the apoptosis rate were the minimum and maximum values previously applied in literature for the same cell phenotypes investigated here (Checa et al., 2011). The value of 10% for the MSCs differentiation rate was selected during the evaluation of the model without mechanics-dependent apoptosis; additionally, a lower value of 5% was tested. Not all combinations of parameters resulted in a model that could be successfully run until the completion of the repair process: the finite element analysis of M5MSC5A15D10 and M15MSC15A5D10 did not reach convergence at day 46 and 35, respectively. In both cases, the issue was ascribed to the differences in MSCs mitosis rate and apoptosis rate. In M5MSC5A15D10, cellular apoptosis might have been too fast compared to the formation of new tissues, as the MSCs differentiation rate was set to the minimum of the investigated values. In M15MSC15A5D10, the low apoptosis rate might have made the model too slow in adapting to the rapid changes in tissue composition consequent to the high MSCs mitosis rate combined to the variations in mechanical environment. Both cases might have generated a too heterogeneous distribution of mechanical properties in the defect, where individual elements resulted too soft compared to the neighbouring ones and were thereby excessively deformed. Thus, MSCs differentiation rate and apoptosis rate were both assigned equal and high values in the subsequent tests, in which no convergence problems were observed. The repair process resulted in comparable outcomes with the investigated variations of cellular mitosis and MSCs differentiation rates (Supplementary Figure 1B). Given the similarities, the set of parameters that resulted in the earliest completion of cellular invasion of the defect was chosen to be employed in all subsequent evaluations (M5MSC15A15D5).


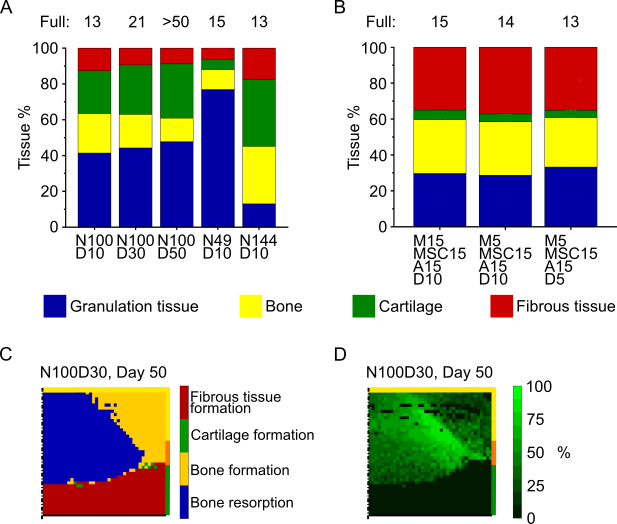


**Supplementary Figure 1.** Sensitivity analysis. A) Influence of maximum number of cells per element and MSCs differentiation rate on tissue formation in model without mechanics-dependent apoptosis; B) influence of mitosis rate and MSCs differentiation rate on tissue formation in model with mechanics-dependent apoptosis. Both plots refer to the tissue percentages at day 50. The day at which the defect was fully populated by cells is reported on top of each column (“Full”). The legend is below the plots; C) representative prediction of tissue formation based on the mechanical stimulus obtained with the model without mechanics-dependent apoptosis; D) exemplary cellular distribution (chondrocytes) corresponding to the same conditions and time of C) and illustrating the mismatch between prediction of tissue formation based on the mechanical stimulus and tissue formation determined by cellular distributions. The black dash-dot lines and the black solid lines mark the axis of symmetry and the articular interface, respectively. The colors of the borders indicate the neighboring tissues in the FE model of the femoral condyle: yellow, orange, and green stand for cancellous bone, subchondral bone, and cartilage, respectively.

# Model with deformable tibial plateau

In the investigated model, the tibia was simplified as a rigid wire. Therefore, the deformable cartilage of the femoral condyle was in contact with a non-deformable interface. However, in the natural joint, the femoral cartilage is in contact with the tibial cartilage, i.e. a deformable interface. To test the influence of the tibial interface on the outcome of the repair process, an additional model featuring a deformable tibial plateau was built (Supplementary Figure 2A). The model with deformable tibia was analogous to the model with rigid tibia, with the only difference that the tibial plateau was modelled as a 2mm-thick cartilage layer upon a rigid wire. The tibial cartilage had a simplified morphology and it was assigned the same material properties as the femoral cartilage (see section 2.1).

At day 1, the octahedral shear strain distribution in the defect was generally higher in the model featuring a deformable tibial plateau (Supplementary Figure 2B right) than in the model featuring a rigid tibial plateau (Supplementary Figure 2B left). Specifically, a more extensive region of high octahedral shear strain with values higher than 30% was found at the interface with the healthy cartilage.

When evaluating the repair process, the higher strain in the model with deformable tibial plateau resulted in the prediction of fibrous tissue formation in the majority of the defect at day 1 (Supplementary Figure 2C top left), in contrast to the prediction of cartilage formation obtained with the rigid tibial plateau (Supplementary Figure 2C bottom left). However, with the progression of the repair process, the prediction of tissue formation in the two models matched. In fact, already at day 10, both models predicted bone growth at the proximal-peripheral corner of the defect, bone resorption in the proximal-central region, cartilage formation in the middle, and fibrous tissue formation at the articular interface (Supplementary Figure 2C right). The main difference was the thicker layer of fibrous tissue predicted in presence of the deformable tibial plateau.

Importantly, when the deformable tibial plateau was implemented, the tibial cartilage came into contact with the softer granulation tissue in the defect. With the progression of the repair process and the formation of stiffer tissues beginning from the proximal base of the defect, a progressively decreasing amount of granulation tissue found itself compressed between stiffer tissues. Such a compression reached high values at day 14, preventing the analysis to converge and thereby interrupting a further evaluation of the repair process.

As there were clear indications that the final outcome of the repair process would not significantly differ with the implementation of a rigid or a deformable tibial plateau, and as the model with rigid tibial plateau presented fewer computational difficulties, the approximation of the articular interface as a rigid interface was considered acceptable and employed in all investigated cases.


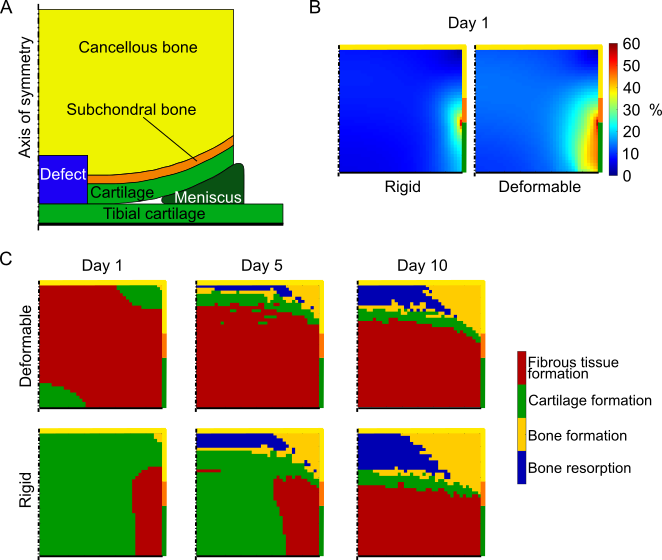


**Supplementary Figure 2.** Model of knee joint featuring a deformable tibial plateau. A) Axisymmetric model of knee with deformable tibial plateau; B) distribution of octahedral shear strain in the defect at day 1 with rigid and deformable tibial plateau (left and right, respectively); C) prediction of tissue formation based on mechanical stimulus at day 1, 5, and 10 (left, middle, and right columns, respectively) with deformable and rigid tibial plateau (top and bottom rows, respectively). The black dash-dot lines and the black solid lines mark the axis of symmetry and the articular interface, respectively. The colors of the borders indicate the neighboring tissues in the FE model of the femoral condyle: yellow, orange, and green stand for cancellous bone, subchondral bone, and cartilage, respectively.
